# Lupeol Stearate Accelerates Healing and Prevents Recurrence of Gastric Ulcer in Rodents

**DOI:** 10.1155/2022/6134128

**Published:** 2022-04-13

**Authors:** Lincon Bordignon Somensi, Philipe Costa, Thaise Boeing, Luísa Nathália Bolda Mariano, Elizama de Gregório, Aline Teixeira Maciel E Silva, Bruna Longo, Claudriana Locatelli, Priscila de Souza, Cássia Gonçalves Magalhães, Lucienir Pains Duarte, Luisa Mota da Silva

**Affiliations:** ^1^Programa de Pós-Graduação em Desenvolvimento e Sociedade (PPGDS), Universidade Alto Vale do Rio do Peixe, CEP, 89500-199 Caçador, SC, Brazil; ^2^Programa de Pós-Graduação em Ciências Farmacêuticas (PPGCF), Núcleo de Investigacões Químico-Farmacêuticas (NIQFAR), Universidade do Vale do Itajaí (UNIVALI), Rua Uruguai, 458, Centro, 88302-202 Itajaí, SC, Brazil; ^3^Departamento de Química, Centro de Ciências Exatas e Naturais, Universidade Federal de Minas Gerais, Belo Horizonte, Minas Gerais, Brazil; ^4^Departamento de Química, Centro de Ciências Exatas e Naturais, Universidade Estadual de Ponta Grossa, Ponta Grossa, Paraná, Brazil

## Abstract

**Objective:**

The focus of this study was to evaluate the gastric healing effect of lupeol stearate (LS) and its ability to minimize ulcer recurrence in rodents.

**Methods:**

To evaluate the gastric healing properties of LS, rats were subjected to 80% acetic acid-induced ulcer model and treated with vehicle, LS (1 mg/kg, p.o.), or omeprazole (20 mg/kg, p.o.), twice daily by seven days. The gastric ulcers were evaluated macroscopically, histologically, and biochemically. To evaluate the effects of LS in gastric ulcer recurrence, mice were ulcerated with 10% acetic acid and treated with vehicle, LS (1 mg/kg, p.o.), or ranitidine (100 mg/kg, p.o.), twice a day for ten days. Then, ulcer recurrence in these animals was induced by IL-1*β* at five days after the treatment period.

**Results:**

The oral treatment with LS accelerated gastric healing by 63% in rats compared to the vehicle group, evidenced by histological improvement and increased gastric mucin levels. Moreover, the gastric healing effects of LS in rats were accompanied by an elevation in glutathione S-transferase activity and a reduction in myeloperoxidase activity. Furthermore, the LS treatment reduced the recurred lesions in mice.

**Conclusions:**

The oral treatment of LS accelerates gastric healing in rats by favoring mucus production and reducing neutrophil migration, and it also can reduce ulcer recurrence. These data highlighted this compound as promising for developing new pharmacological strategies for the management of gastric ulcer.

## 1. Introduction

The incidence of stomach diseases is high worldwide, impacting human health worldwide. Gastric ulcers are lesions in the stomach's mucosa, giving it vulnerability to bleeding or perforation, which eventually aggravates the primary disease [1]. Gastric ulcers occur due to an imbalance between the protective and aggressive factors in the gastric mucosa. Some exogenous factors lead to this imbalance, such as excessive alcohol consumption, prolonged treatment with nonsteroidal anti-inflammatory drugs (NSAIDs), infection with *Helicobacter pylori*, and social stress due to the modern lifestyle [2]. Given the potentiality of gastric acid secretion and pepsin as endogenous aggressors, the treatment of gastric ulcers is often based on gastric acid suppression using histamine two receptor antagonists (H2-RAS) such as ranitidine, or proton pump inhibitors (IBP) such as omeprazole [3, 4].

Since the discovery of omeprazole almost 40 years ago, the therapeutic resources aim to reduce gastric acidity but not strengthen the mucosal protective factors, such as the mucus and bicarbonate barrier, cell proliferation, proper blood flow, or antioxidant defenses. However, prolonged antisecretory therapy possesses several adverse effects, including a high risk of gastric cancer in patients infected by *H. pylori* [2, 5, 6]. Another issue is the poor quality of gastric healing achieved by IBP therapy, which favors the ulcer recurrence mainly after the therapy interruption, leading the patient to a prolonged treatment time, increasing the chances of the adverse effect [6].

One of the problems related to the gastric ulcer is its capacity to recurrence [7], a complex process involving increased levels of inflammatory mediators, such as tumor necrosis factor (TNF) and interleukin (IL)-1*β* at the gastric mucosa [8]. This increase in inflammatory cytokines also contributes to oxidative stress, amplifying the lesion in the gastric mucosa [9]. Therefore, the basis for the treatment of gastric ulcers may be associated with compounds able to minimize proinflammatory factors and oxidative damage in gastric tissues.

Lupeol is a much studied pentacyclic triterpene found in several plants and with gastroprotective activity already described [10]. Moreover, this triterpene has several biological activities, including in vitro [11] and in vivo [12] anticancer properties, antidiabetic effects [13], topical anti-inflammatory actions [14], and antimalarial potency [15], besides has been a candidate as a nonhormonal male contraceptive [16]. Recently, through the esterification of lupeol, it was possible to obtain lupeol stearate (LS) [17], a derivative with potent gastroprotective activity [18].

Indeed, the confirmation of the potent gastroprotection provided by LS in acute gastric ulcer models through a preventive and nontherapeutic approach by our research group has shed light on new questions regarding the antiulcer potential of this ester. Therefore, this study aimed to evaluate the therapeutic efficacy of oral treatment with LS in gastric ulcer healing and its ability to reduce ulcer recurrence in rodents.

## 2. Materials and Methods

### 2.1. Obtaining Lupeol Stearate (LS) and Choice of Dose

Lupeol was isolated from *Maytenus salicifolia* Reissek (Celastraceae) hexanic extract as described in detail by Magalhães et al. [19] and esterification with appropriate reagents gave rise to LS was performed as described in detail by Silva et al. [17]. The oral gastroprotective dose of LS of 1 mg/kg was chosen for this study based on findings from Somensi et al. [18].

### 2.2. Animals

Male Wistar rats (200–250 g) and male Swiss mice (25–30 g) were obtained from the Universidade do Vale do Itajaí and kept in polypropylene boxes at 22 ± 2°C in 12-hour light-dark cycles with free access to water and feed. The animals were deprived of food eight hours before the experiments. All protocols were approved by the Institutional Committee on Animal Ethics of UNIVALI (CEUA/UNIVALI, approval number 056/2017 and 15/15p), conducted according to the ARRIVE guidelines, and following the International Standards and Ethical Guidelines on Animal Welfare.

### 2.3. Chronic Ulcer Induced by 80% Acetic Acid in Rats

The acetic acid-induced ulcer was performed in rats as described by Okabe [20], with few modifications. The rats were intraperitoneally anesthetized with xylazine and ketamine (10 mg/kg, and 50 mg/kg, respectively), and a laparotomy was performed to expose the stomach serosa. Then, 500 *μ*L of 80% acetic acid were instilled into the gastric serosa using a plastic cylinder (6 mm diameter) to induce the ulcers. The ulcer induction was performed with 80% acetic acid instead of 100% as described by Okabe [20], given the frequency of animals that suffered gastric perforation and died. Therefore, to refine the method in our experimental conditions, we applied acetic acid solution to the serosa of the stomach at a concentration of 80% in all experimental groups. After 1 min, the acid was aspirated, the serosa was washed with 0.9% saline solution, the stomach was carefully relocated to the abdominal cavity, and the incision was sutured. After recovery, the animals were randomly divided into different groups (*n* = 8). On the second day after ulcer induction, they were orally treated with vehicle (10% dimethyl sulfoxide, DMSO), 1 mL/kg, per os (p.o.), omeprazole (20 mg/kg, p.o.) or LS (1 mg/kg, p.o.), twice a day, for seven days. In addition, one group of animals was not submitted to ulcer induction and named naïve group, which was used to allow comparison of the treatment groups to a nonulcerated group. At the end of the treatment period, the animals were euthanized in a CO_2_/O_2_ chamber, the stomach was removed, and the gastric ulcer area (mm^2^) was measured using a ruler [20].

### 2.4. Histological and Histochemical Evaluation

Histological and histochemical procedures were performed following the same protocols used by Da Silva et al. [21] and Somensi et al. [22]. The ulcer site was embedded in a fixative solution (85% alcohol, 10% formalin, and 5% acetic acid). Then, the samples were soaked in paraffin and cut into sections of 5 *μ*m. A part of the histological slices was stained by hematoxylin and eosin, while the other part was submitted to the Schiff Periodic Acid histochemical method to measure the amount of Periodic acid-Schiff (PAS)-stained mucin-like glycoproteins using ImageJ® software.

### 2.5. Preparation of the Subcellular Fraction and Quantification of Proteins

Following the same protocols used by Da Silva et al. [21], the acetic acid-ulcerated gastric mucosa was homogenized with 200 mM potassium phosphate buffer (pH 6.5). The homogenate was used to measure reduced glutathione (GSH) levels. After the homogenate was centrifuged at 9000 × *g* for 20 minutes and the supernatant was used to evaluate the activity of glutathione S-transferase (GST), superoxide dismutase (SOD), and catalase (CAT), the precipitate was used to measure the activity of myeloperoxidase (MPO). The protein concentrations were determined in all samples using Bradford reagent and bovine albumin as standard, following the manufacturer's instructions (Bio-Rad®, CA, USA).

### 2.6. Quantification of GSH Levels

To quantify the GSH levels, 50 *μ*L of homogenate were deproteinized with 40 *μ*L of 12.5% trichloroacetic acid and centrifuged at 4000 × *g* by 15 min at 4°C. After the centrifugation, 20 *μ*L of supernatant was added to 270 *μ*L of TRIS buffer (pH 8.9) plus 10 *μ*L of 5,5′-ditiobis-2-nitrobenzoic acid and incubated for five minutes. The absorbance of this reaction medium was measured at 415 nm. To GSH quantification, the values were interpolated in a standard GSH curve (1.25–10.00 *μ*g/mL), and the results were expressed in *μ*g/mg of tissue as described by Sedlak et al. [23].

### 2.7. Determination of SOD, CAT, and GST Activity

The determination of SOD activity was made as previously demonstrated by Marklund and Marklund [24], where samples of the supernatant (20 *μ*L) were incubated with 200 mM Tris-HCl-EDTA (pH 8.5) and 1 mM pyrogallol for 20 min. Thereafter, the absorbance was measured at 405 nm, and the SOD activity was expressed as U/mg of protein.

The CAT activity was determined as described by Aebi [25]. In this test, an aliquot of the supernatant (5 *μ*L) was added to 295 *μ*L of the reaction medium composed of 200 mM Tris-HCl-EDTA (pH 8.5) plus 47.35 mL of ultrapure water and 172.5 *μ*L of 30% H_2_O_2_. Then, the decrease in the absorbance of this reaction mixture was immediately measured at 240 nm, and the results were expressed in *μ*mol/min/mg of protein.

GST activity was measured as described by Habig et al. [26], which 50 *μ*L of the supernatant was incubated with 250 *μ*L of a reaction medium composed by 1 mM 1-chloro-2,4-dinitrobenzene plus 1 mM GSH in phosphate buffer (pH 6.5), the increase in the absorbance was measured at 340 nm, and results were expressed in *μ*mol/min/mg of protein.

### 2.8. Determination of MPO Activity

The precipitate was obtained as described in section 2.5 and was used to determine the MPO activity proposed by Bradley et al. [27] and Young et al. [28]. For this analysis, samples were resuspended in 80 mM (pH 5.4) potassium phosphate buffer containing 0.5% hexadecyl trimethylammonium bromide and recentrifuged at 11000 × *g* for 20 min at 4°C. The MPO activity was determined by reaction between the supernatant and H_2_O_2_ plus 3,3′,5,5′-tetramethylbenzidine. The absorbance of this reaction was registered at 620 nm, and the results are expressed in units of milli optical density (mO.D)/mg of protein.

The in vitro activity of MPO was verified using a homogenate obtained from the stomach of an ulcerated mouse treated with a vehicle, that is, with known high MPO activity. The supernatant samples were incubated with LS (0.1 to 1000 *μ*g/mL) at 25°C for 15 minutes. The MPO activity was measured as described above, and the results were expressed in the same manner.

### 2.9. 10% Acetic Acid-Induced Gastric Ulcer Recurred by IL-1*β* Administration in Mice

First, the acetic acid-induced ulcer was performed as described by Okabe et al. [20] with modifications to mice. Mice were anesthetized with xylazine and ketamine (10 mg/kg and 50 mg kg, i.p, respectively). The abdominal wall was opened, the stomach exposed, and a plastic cylinder (2 mm diameter) was applied to the serosa to instill 10% acetic acid. After 1 min, the acetic acid was aspirated and replaced by 0.9% saline solution. The saline solution was aspirated, the cylinder was removed from the serosa, the stomach was replaced, and the abdominal wall was sutured. After recovery from anesthesia, the mice were randomly divided into groups (*n* = 6) and treated with vehicle (1% DMSO, 10 mL/kg, p.o), ranitidine (100 mg/kg, p.o), or LS (1 mg/kg, p.o). The treatments were started on the second day after the surgery was performed twice a day for ten days. After the oral treatment period, the animals did not receive treatment from the 11th to 14th day after ulcer induction. After this, interleukin (IL)-1*β* (1 *μ*g/kg) was administered by an intraperitoneal route on the 15th day after ulcer induction to elicit ulcer recurrence as described by Watanabe et al. [8] with modifications to mice. A group of acetic acid-ulcerated mice treated with vehicle received saline instead of IL-1*β*. After 24 hours, the animals were euthanized in a CO_2_/O_2_ chamber. After, the stomach was removed, and the recurred ulcer area (mm^2^) was measured using a ruler.

### 2.10. Statistical Analysis

The data obtained were presented as means ± standard error of means (S.E.M.). One-way or two-way analysis of variance (ANOVA) followed by the Bonferroni was employed. Analyses were performed using the Program for Windows, GraphPad Prism version 6.0 (GraphPad software, San Diego, USA). A *p* < 0.05 was adopted as statistically significant in all experiments.

## 3. Results

### 3.1. LS Promotes the Healing of Gastric Mucosa of Rats

**Figure 1:**
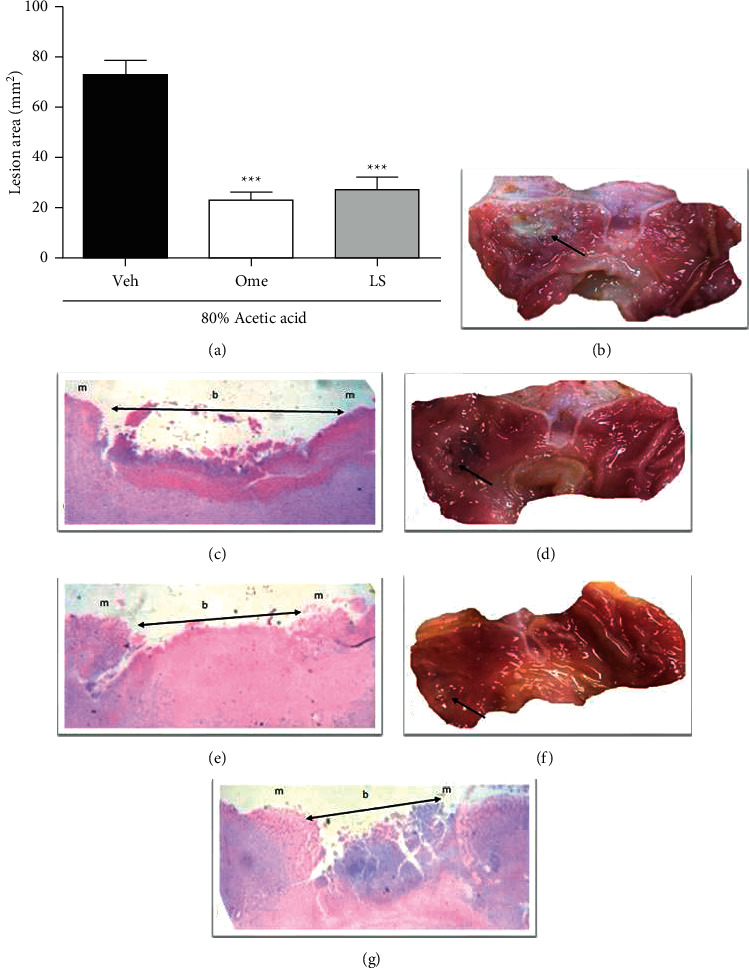
Oral administration of lupeol stearate (LS) promotes gastric healing activity in rats. The rats were orally treated with vehicle (Veh: 10% DMSO, 1 mL/kg), omeprazole (Ome: 20 mg/kg), or LS (1 mg/kg) twice a day, by 7 days, after the gastric ulcer induction. Panel (a) shows the area of the gastric ulcer (mm^2^), and results are expressed as means ± S.E.M. (*n* = 8) analyzed using one-way ANOVA followed by Bonferroni's test with ^*∗∗∗*^*P* < 0.001 compared to Veh group. Representative macroscopic images of gastric mucosa from ulcerated rats treated with vehicle, omeprazole, or LS are shown in panels (b), (d), and (f), respectively, with black arrows indicating the ulcer site. Histological hematoxylin/eosin appearance of ulcer site from ulcerated rats treated with vehicle, omeprazole or LS are shown in panels (c), (e), and (g), respectively, where m indicates the margin of ulcer and b indicates the ulcer base.

As observed in Figure 1, the treatment with LS (1 mg/kg p.o.) or omeprazole (20 mg/kg p.o.) twice a day for seven days reduced the lesion area of acetic acid-induced ulcer by 63% and 68%, respectively, when compared to the vehicle-treated group (73.33 ± 5.50 mm^2^, Figure 1(a)). The data from this macroscopic evaluation were confirmed in histological analysis at the ulcer site, where the ulcerated-vehicle group showed deep damage characterized by an extensive ulcer base and a little epithelium bordering this base and composing its margin (Figures 1(b) and 1(c)). In contrast, the treatment with omeprazole (Figures 1(d) and 1(e)) or LS (Figures 1(f) and 1(g)) increased the ulcer margin through a re-epithelialization and decreased the ulcer base, suggesting an acceleration in the healing process, compared to the ulcerated vehicle group.

### 3.2. LS Elevated the Periodic Acid-Schiff (PAS)-Stained Mucin-Like Glycoproteins in Ulcerated Gastric Mucosa

**Figure 2:**
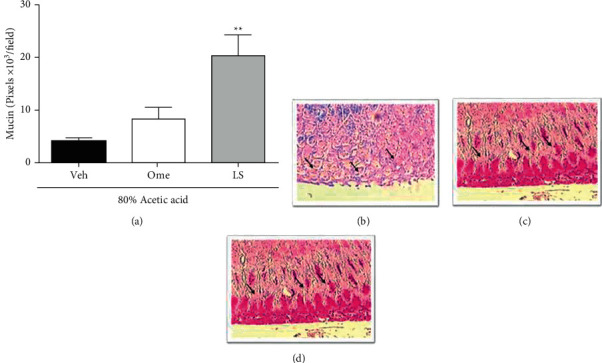
Lupeol stearate (LS) elevated staining for mucin-like glycoproteins in ulcerated gastric mucosa of rats. The rats were orally treated with vehicle (Veh: 10% DMSO, 1 mL/kg), omeprazole (Ome: 20 mg/kg), or LS (1 mg/kg) twice a day, by 7 days, after the gastric ulcer induction. Panel (a) shows the quantification of PAS-staining, and results are expressed as means ± S.E.M. (*n* = 8) analyzed using one-way ANOVA followed by Bonferroni's test with ^*∗∗*^*P* < 0.01 compared to the Veh group. Panels (b–d) are representative images of groups orally treated with vehicle, omeprazole, or LS. Panels (b–d): magnification = 400x.

The staining of mucin-like glycoproteins using a histochemical technique is shown in Figure 2(a), where the administration of LS (1 mg/kg, p.o) but not omeprazole (20 mg/kg, p.o), raised the PAS-stained mucin-like glycoproteins by 391% when compared to the ulcerated group that received vehicle (4.12 ± 0.6 pixels × 103/field, Figure 2(a)). Representative images of the PAS-stained mucin-like glycoproteins labeling from ulcerated groups treated with vehicle, omeprazole, or LS can be verified in Figures 2(b)–2(d), respectively.

### 3.3. LS Did Not Change GSH Levels but Increased GST Activity at the Ulcer Site

As shown in Table 1, the ulcerated group treated with vehicle showed a reduction of 73% in the GSH levels and 65% in the GST activity at the ulcer site, compared to the nonulcerated group (naive group: 618.5 ± 98.1 *μ*g of GSH/mg of tissue and 119.4 ± 20.9 *μ*mol/min/mg of protein, respectively). Although the treatments with omeprazole (20 mg/kg, p.o) or LS (1 mg/kg, p.o) did not avoid the GSH, they did enhance the GST activity by 332% and 278%, respectively, when compared to the ulcerated group treated with vehicle (41.7 ± 20.8 *μ*mol/min/mg of protein).

### 3.4. LS Did Not Change the SOD and CAT Activities but Reduced the Activity of MPO at the Ulcer Site

The SOD activity was increased by 105% in the ulcerated group treated with vehicle compared to the nonulcerated group (naive: 3.77 ± 0.21 U SOD/mg of protein). However, the groups treated with omeprazole (20 mg/kg, p.o) or LS (1 mg/kg, p.o) not showed changes in SOD activity compared to the ulcerated group treated with vehicle (Table 1). Furthermore, the CAT activity was reduced by 40% in the ulcerated group treated with vehicle concerning the nonulcerated group (naive: 223 ± 22 *μ*mol/min/mg of protein). The group treated with omeprazole (20 mg/kg, p.o) presented an increase of 30% in the CAT activity, but the treatment with LS (1 mg/kg, p.o) did not change the CAT activity, compared to the ulcerated group treated with vehicle (Table 1).

In addition, the MPO activity was elevated by 287% in the ulcerated group treated with vehicle compared to the nonulcerated group (naive: 1.16 ± 0.13 mD.O/mg of protein). In contrast, the treatment with omeprazole (20 mg/kg, p.o) or LS (1 mg/kg, p.o) reduced the MPO activity by 42% and 32%, respectively, compared to the ulcerated group treated with vehicle (Table 1).

Given the decrease in MPO activity in rats treated with LS, the subsequent measurement evaluated whether the reduction in neutrophil migration caused this reduction to the ulcer site or by direct inhibition of the MPO enzyme. Thus, a homogenate sample obtained from ulcerated tissue (vehicle-treated animal) was incubated with LS (0.1–1000 *μ*L/mL), and MPO activity was measured again. Interestingly, LS 100 *μ*L/mL inhibited the MPO activity by 26% in this assay, proving that LS can weakly directly inhibit the MPO enzyme and reduce neutrophil-mediated ROS formation (data not shown).

### 3.5. LS Reduced the Recurrence of 10% Acetic Acid-Induced Gastric Ulcer Recurred by IL-1*β* Administration in Mice

**Figure 3:**
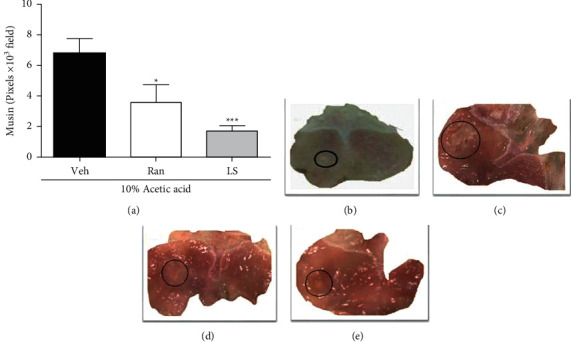
Lupeol stearate (LS) reduced the ulcer recurrence induced by IL-1*β* in mice. The animals were orally treated with vehicle (Veh: 10% DMSO, 1 mL/kg), ranitidine (Ran: 100 mg/kg), or LS (1 mg/kg) twice a day, by 10 days, after the gastric ulcer induction. To induce ulcer recurrence, IL-1*β* (1 *μ*g/kg, i.p) was given the 15th day after ulcer induction. Panel (a) shows the area of the gastric ulcers (mm^2^), and the results are expressed as means ± S.E.M. (*n* = 8) analyzed by one-way ANOVA followed by Bonferroni's test with ^*∗*^*P* < 0.05; ^*∗∗∗*^*P* < 0.001, compared to Veh group. Representative images of gastric mucosa from ulcerated rats treated with the vehicle without IL-1*β* exposure, vehicle with IL-1*β* exposure, ranitidine with IL-1*β* exposure, or LS with IL-1*β* exposure are shown in panels (b), (d), (f), and (e), respectively, with circles indicating the ulcer site.

As shown in Figure 3(a), mice exposed to 10% acetic acid-induced gastric ulcer recurred by IL-1*β* administration and treated with vehicle showed a unique gastric lesion compatible with ulcers morphology in an extension of 6.8 ± 0.9 mm^2^ at the 15th day after the ulcer induction, indicating recurrence of the lesion because there was no ulcerative lesion in the gastric mucosa in ulcerated mice that did not receive IL-1 3*β* on the same day after ulcer induction. The group treated with ranitidine or LS (1 mg/kg, p.o) showed a reduction of 48% and 78% in the severity of gastric ulcer recurrence, respectively. It is worth mentioning that 25% of the animals in the group treated with LS (1 mg/kg, p.o) did not present an evident lesion. Representative images of 10% acetic acid-ulcerated mice treated with vehicle and not exposed to IL-1*β* are shown in Figure 3(b). In contrast, the macroscopic appearance of ulcer recurrence from mice exposed to IL-1*β* and treated with vehicle, ranitidine, and LS are depicted in Figures 3(c), 3(d), and 3(e), respectively.

## 4. Discussion

In previously experimental investigations about the antiulcer potential of lupeol stearate (LS), our research group showed that this compound promotes a potent gastroprotective effect against gastric lesions induced by ethanol in rodents [18]. Given those results, the present study evaluated the capacity of LS to promote gastric healing in the acetic acid-induced ulcer model, which highly resembles human gastric ulcers and can relapse [29]. Indeed, it was possible to show that LS favors gastric healing, mainly due to enhancing the mucus barrier. It can reduce ulcer recurrence, especially by preventing inflammation mediated by neutrophils.

It is well defined that the acetic acid instillation on the gastric serosa produces a mucosal lesion that resembles humans in location, chronicity, severity, and the healing process [30]. The healing process of these gastric ulcers is extremely complex because it involves the migration and multiplication of epithelial cells that are located at the margin of the ulcer, which reestablishes the glandular disposition and stimulates angiogenesis at the base of the lesion through the stimulation of granulation tissue [21, 31]. Extensive ulcerative lesions were observed macroscopically in the ulcerated group treated with the vehicle but not in the group treated with 1 mg/kg of LS daily.

Furthermore, deep ulcerative damage characterized by a larger ulcer base with little proliferating epithelium bordering this base was found in the histological slices from rats that received vehicle. In contrast, the histological examination of ulcer site from rats treated with LS could verify a contraction in ulcer base due to the ulcer margin growth, which reflects a more advanced re-epithelialization process than in animals treated with vehicle. In agreement with these features, Beserra et al. [32] showed that lupeol possesses wound healing potential in hyperglycemic conditions and may be helpful as a treatment for chronic wounds in diabetic patients. Moreover, studies with oleanolic acid, a triterpene structurally related to lupeol, have proven its gastric healing actions [33, 34].

Some experimental studies have shown that gastric ulcers induced by acetic acid have a multifactorial process that begins with the depletion of the mucous content of the stomach wall, associated with excessive production of free radicals in consequence to an increase in the proinflammatory interleukins TNF-*α*, IL-1*β*, and IL-6, accompanied by an increase in neutrophil infiltration into the gastric mucosa [21, 35–37]. For this reason, the effect of LS on mucin levels, gastric antioxidant defenses, and neutrophil migration was investigated in this study.

Classically, the mucin levels in the gastrointestinal tract have been measured using the PAS-staining method, which stains the neutral mucins bright magenta. Our results showed that the treatment with LS enhanced the labeling for Periodic acid-Schiff (PAS)-stained mucin-like glycoproteins, mainly inside the glands. The gel-forming mucins covering the gastric mucosa are heavily glycosylated glycoproteins that form a barrier between the stomach and the external environment [38] and are strongly implicated in the gastric healing process because they protect the epithelial cells during the regenerative process [39]. In this way, it is possible to infer that the gastric healing effects of LS in the ulcerated tissue are firstly mediated by the increase in this protective barrier, consequently creating a favorable microenvironment for the recovery of that mucosa.

In agreement with these findings, our research group's previous results using a model of indomethacin-induced ulcers evidenced an increase in PGE2 levels after the treatment of ulcerated rodents with LS [18]. Given that PGE2 mediates mucin production, it is reasonable to infer that the enhancement in this eicosanoid in the gastric mucosa promoted by LS increases the mucin levels also observed in this study.

Apart from mucins, the wound healing capacity of the gastrointestinal tract also depends on the balance in the redox state of the mucosa. Indeed, research with antioxidants has shown that they can interfere with the ulcerative process into the gastric mucosa [40], and the capability of LS to enhance the antioxidant defenses in the stomach was already evidenced by our research group using acute ulcer models [18]. Because of this, we evaluated the levels or activity of antioxidative resources at the site of acetic acid-induced ulcers from rats treated or not with LS.

GSH is among the antioxidants with higher levels (7–8 mM) in the gastric mucosa compared to other parts of the gastrointestinal tract, providing additional protection against gastric injuries. Reduced levels of GSH were found in the acetic acid-induced ulcers in rats treated with vehicle, like our previous study [21]. The oral treatment with LS did not prevent such depletion in contrast to previous results using ethanol-induced acute ulcers [18]. It is worth noting that the difference in the nature of the ulcerative lesions between both studies is directly related to the differences in the mode of action by which LS maintains the integrity of the gastric mucosa.

As revised by Pérez et al. [41], the enzymes glutathione S-transferases (GSTs), superoxide dismutases, catalase, and glutathione peroxidases exert a tremendous protective role in the gastrointestinal epithelium against injury and inflammation. In the meantime, our results showed that the oral treatment with LS increased the GST activity suggesting that the compound helps in the detoxification of the ulcerated mucosa. Interestingly, it is possible that the increase in GST activity promoted by LS may be related to the reduced GSH levels since the detoxifying effects of this enzyme occur at the expense of GSH oxidation because this tripeptide is an important GST cofactor.

In addition to GST, SOD and CAT are part of the enzymatic antioxidant defenses in the gastric mucosa. SOD causes the dismutation of the superoxide anion to hydrogen peroxide, while CAT converts hydrogen peroxide into water and oxygen [42]. In the ulcerated mucosa from rats treated with the vehicle was possible to observe that while SOD activity was increased, CAT activity was reduced, suggesting that there was oxidative damage mediated by hydroperoxides in these animals. Unexpectedly, this imbalance in the activity of both enzymes was also found in the ulcerated mucosa of animals treated with omeprazole or LS. Both compounds were able to promote gastric healing in this study; in the case of omeprazole, the mechanism is recognized. The inhibition of the proton pump reduces gastric acidity so that it favors healing even without adjacent antioxidants mechanisms. In contrast, LS does not reduce gastric secretion [18], and other adjacent mechanisms, including the increase in mucus barrier, can explain its gastric healing effects even without needing these antioxidant routes.

It is known that inflammation is a crucial component during the ulcerative process at the gastric mucosa, and neutrophils contribute mostly to this damage [43]. The neutrophil migration has been indirectly measured by the MPO activity because this enzyme is found in the azurophil granules inside neutrophils [44]. Therefore, MPO activity has been used in some studies as an index of neutrophil infiltration [21, 45, 46] and being able to produce large amounts of harmful free radicals in the gastric mucosa [44]. Indeed, our results confirmed the increase in MPO activity at the ulcers site of the ulcerated group treated with the vehicle. On the contrary, the treatment with LS reduced this parameter in the ulcerated tissue, being possible to infer that the inflammatory process mediated by neutrophil migration was minimized due to the treatment with LS. These results agree with those found using the ethanol-induced ulcer model, where the pretreatment with LS also reduced the MPO activity [18].

In addition, the incubation with LS (100 *μ*L/mL) inhibited the MPO activity directly by 26%, suggesting that in addition to the reduction in neutrophil migration, this triterpene also has inhibitory activity on the ability of this enzyme to produce reactive oxygen species. Corroborating with our data, the inhibitory actions of lupeol in neutrophils were already described by Yamashita et al. [47], mainly its ability to suppress superoxide generation by preventing tyrosyl phosphorylation of a 45.0-kDa protein in human neutrophils.

Considering the results discussed so far, we were interested in knowing the effect of LS administration (1 mg/kg) on ulcer recurrence. To answer this question, the model of 10% acetic acid-induced gastric ulcer recurred by IL-1*β* administration was reproduced in this study in mice. IL-1*β*, produced mainly by inflammatory cells such as monocytes/macrophages, plays a role in many inflammatory processes [48]. Moreover, the production of several cytokines, including IL-1*β*, in gastric mucosa is increased in subjects infected with *H. pylori*, and this infection has been associated with ulcer recurrence [8]. Therefore, Watanabe et al. [8] developed a recurrence ulcer model in which the administration of IL-1 beta can cause recurrence of experimental gastric ulcers in rats, and neutrophilic infiltration into scarred mucosa is responsible for this recurrence. In this model, adapted for mice, our results showed that animals previously treated with LS presented a lower degree of reappearance of the gastric lesion after 24 hours of IL-1*β* administration. There was no evident lesion in 25% of this group (result found in triplicate of experiments). In agreement, a study with triterpenes reveals that these compounds modulate the production of ROS in the microenvironment of the wound, accelerating the process of tissue repair, since inducing cell proliferation, cell migration, and collagen deposition [49]. This result shows the quality of healing promoted by LS and indicates how promising this compound can be as a treatment for gastric ulcers in patients with high recurrence levels.

## 5. Conclusion

These results confirm that lupeol stearate (LS) has gastric healing activity in an already ulcerated mucosa. Its mode of action involves the increase in mucin production, the reduction in neutrophil migration, and the favoring of GST activity. These LS actions reached an excellent quality of healing and avoided a recurrence of the ulcer even in the face of an inflammatory stimulus. Therefore, our findings suggest that LS may serve as a novel therapeutic option to treat gastric ulcers and their recurrence by regulating gastric protective factors and reducing the inflammatory process.

## Figures and Tables

**Table 1:** Effects of lupeol stearate (LS) on biochemical parameters at the ulcer site.

	MPO (mD.O/ mg protein)	GSH (*μ*g/mg tissue)	SOD (U/mg protein)	CAT (*μ*mol/ min/ mg protein)	GST (*μ*mol/min/mg protein)
Naive	1.16 ± 0.13	618.5 ± 98.1	3.77 ± 0.21	223 ± 22.4	119.4 ± 20.9
Vehicle (1 ml/kg, p.o)	4.49 ± 0.68^a^	169.6 ± 16.5^a^	7.72 ± 0.34^a^	132.3 ± 37.4	41.7 ± 20.8^a^
Omeprazole (20 mg/kg, p.o)	2.59 ± 0.31^b^	210.8 ± 66.7^a^	8.28 ± 0.27^a^	302.9 ± 53.7^b^	180.2 ± 27.3^b^
LS (1 mg/ kg, p.o)	3.07 ± 0.35^ab^	243.8 ± 51.7^a^	7.16 ± 0.15^a^	70.3 ± 25.5^a^	157.8 ± 21.8^b^

MPO, myeloperoxidase; GSH, reduced glutathione; SOD, superoxide dismutase; CAT, catalase; GST, glutathione S-transferase (GST *μ*mol/ min/ mg protein). Values expressed in mean ± E.P.M (n = 6). One-way ANOVA followed by Bonferroni's test (*n* = 8). ^a^*P* < 0.05 when compared to the naïve (nonulcerated) group. ^b^*P* < 0.05 when compared to the ulcerated-vehicle group.

## Data Availability

The data that support the findings of this study are available from the corresponding author upon reasonable request.
